# LVCA-Net: Lightweight LiDAR Semantic Segmentation for Advanced Sensor-Based Perception in Autonomous Transportation Systems

**DOI:** 10.3390/s26010094

**Published:** 2025-12-23

**Authors:** Yuxuan Gong, Yuanhao Huang, Li Bao, Jinlei Wang

**Affiliations:** 1 School of Aviation, Inner Mongolia University of Technology, Hohhot 010051, China; 20221100526@imut.edu.cn (Y.G.);; 2 State Key Lab of Intelligent Transportation System, Beihang University, Beijing 100191, China; 3 School of Transportation Science and Engineering, Beihang University, Beijing 100191, China

**Keywords:** intelligent machines, deep learning, LiDAR semantic segmentation, lightweight 3D networks, coordinate-guided attention, autonomous driving

## Abstract

Reliable 3D scene understanding is a fundamental requirement for intelligent machines in autonomous transportation systems, as on-board perception must remain accurate and stable across diverse environments and sensing conditions. However, LiDAR point clouds acquired in real traffic scenes are often sparse and irregular, and they exhibit heterogeneous sampling patterns that hinder consistent and fine-grained semantic interpretation. To address these challenges, this paper proposes LVCA-Net, a lightweight voxel–coordinate attention framework designed for efficient LiDAR-based 3D semantic segmentation in autonomous driving scenarios. The architecture integrates (i) an anisotropic depthwise residual module for direction-aware geometric feature extraction, (ii) a hierarchical LiteDown–LiteUp pathway for multi-scale feature fusion, and (iii) a Coordinate-Guided Sparse Semantic Module that enhances spatial consistency in a cylindrical voxel space while maintaining computational sparsity. Experiments on the SemanticKITTI and nuScenes benchmarks demonstrate that LVCA-Net achieves 67.17% mean Intersection over Union (mIoU) and 91.79% overall accuracy on SemanticKITTI, as well as 77.1% mIoU on nuScenes, while maintaining real-time inference efficiency. These results indicate that LVCA-Net delivers scalable and robust 3D scene understanding with high semantic precision for LiDAR-only perception, making it well suited for deployment in autonomous vehicles and other safety-critical intelligent systems.

## 1. Introduction

Deep learning has become a core driver in the development of intelligent machines, enabling autonomous systems to perceive, reason, and act robustly in complex real-world environments. In the broader context of intelligent transportation and autonomous driving, recent surveys summarize key milestones in perception, decision-making, and system integration, underscoring the rapid evolution of intelligent vehicles [1,2]. Within this landscape, LiDAR-based 3D semantic segmentation plays a central role because it provides dense, geometry-aware scene representations that support downstream functions such as autonomous navigation, motion prediction, safety monitoring, and high-level scene reasoning. Compared with RGB-based perception, LiDAR provides accurate, illumination-invariant range measurements, making it indispensable for robust scene understanding in intelligent vehicles and mobile robotic platforms [3,4,5,6,7].

**Figure 1:**
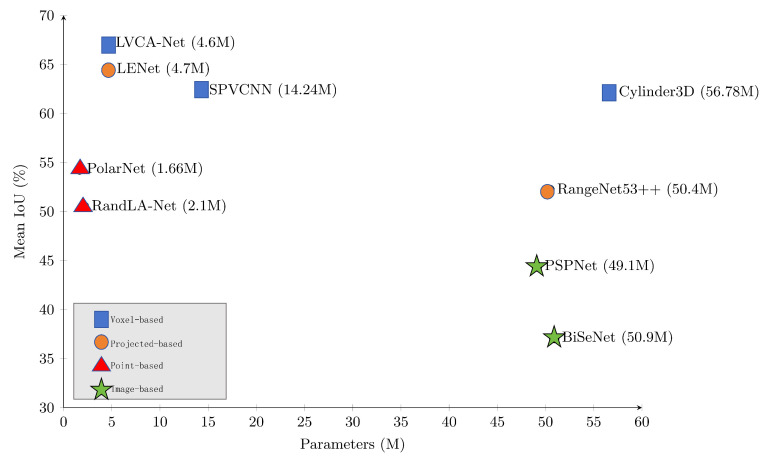
Accuracy, parameter size, and inference speed of 3D LiDAR semantic segmentation models on SemanticKITTI.

Despite rapid progress, achieving both high accuracy and real-time LiDAR segmentation on intelligent machines remains highly challenging. Outdoor LiDAR scans are large-scale, irregular, and highly sparse. Processing them at high spatial resolution or across multiple frames increases computational demand, memory footprint, and end-to-end latency, which conflicts with the real-time requirements of autonomous systems [8,9,10,11,12]. As shown in Figure 1, existing models lie along an accuracy–efficiency spectrum: high-accuracy networks are often computationally heavy, whereas lightweight designs may sacrifice geometric detail and recognition performance to satisfy runtime constraints.

Current 3D semantic segmentation approaches can be grouped into several major families. Point-based methods [13,14,15] preserve geometric fidelity but face scalability bottlenecks on large-scale outdoor scans. Voxel-based networks such as MinkUNet [16] and Cylinder3D [17] improve computational regularity on modern hardware but require careful resolution control to avoid prohibitive cost. Hybrid approaches, including SPVCNN [18] and RangeViT [19], attempt to balance contextual reasoning and efficiency but often incur additional memory usage and training complexity.

In voxel-based systems, discretization introduces additional challenges. Coarse voxel partitions can break object continuity, causing points from a single instance to fall into separate voxels and resulting in label discontinuities. Conversely, thin structures and mixed-occupancy voxels introduce semantic ambiguity when different objects are aggregated into the same voxel. Increasing voxel resolution can alleviate quantization artifacts but leads to rapid growth in memory consumption and computational cost, which is incompatible with the constraints of embedded intelligent machines [16,17].

Long-range perception further benefits from multi-frame fusion [20,21], while cross-dataset deployment remains difficult due to variations in LiDAR density and scene composition across datasets such as SemanticKITTI [22] and nuScenes [23]. Collectively, these factors exacerbate the challenge of achieving both high accuracy and real-time performance.

In addition to application-oriented surveys in autonomous driving and intelligent transportation, recent comprehensive reviews have systematically summarized the algorithmic landscape of 3D point cloud semantic segmentation. Xie et al. [24] provide an early taxonomy covering point-based, voxel-based, and multi-view projection methods. More recent surveys, such as Yang et al. [25] and Betsas et al. [26], further refine these categories by discussing sparse convolutional networks, hybrid fusion architectures, transformer-based designs, and lightweight segmentation strategies. These review articles suggest that voxel-based sparse convolution remains a dominant paradigm for scalable LiDAR perception, while attention mechanisms are increasingly integrated to enhance geometric reasoning. Building on this line of research, LVCA-Net adopts a voxel-based representation and incorporates coordinate-guided attention to achieve lightweight yet expressive semantic segmentation for autonomous driving scenarios.

To address these challenges, we propose LVCA-Net, a lightweight voxel–coordinate attention framework designed for intelligent machines that require fast and reliable 3D perception. LVCA-Net integrates an anisotropic depthwise residual module for efficient direction-aware geometric feature extraction, a hierarchical LiteDown–LiteUp path for multi-scale feature refinement, and a Coordinate-Guided Sparse Semantic Module for modeling long-range dependencies within cylindrical voxel space. The architecture maintains a fully sparse pipeline to control computational cost while preserving structural detail through cylindrical partitioning and point-level refinement. Experiments on SemanticKITTI and nuScenes show that LVCA-Net achieves an improved balance between accuracy and latency, placing it in a favorable region of Figure 1, and further demonstrates robustness to noise, temporal fusion, and cross-domain transfer. Throughout this work, the methodology, experiments, and conclusions are strictly confined to LiDAR-based 3D semantic segmentation without incorporating multimodal fusion.

## 2. Related Work

We review prior work along three threads that underpin our design: (i) the landscape of point cloud segmentation with an emphasis on voxel discretization, (ii) lightweight 3D network design, and (iii) 3D attention mechanisms with coordinate priors. For completeness, we also cite representative projection-based and image-based approaches.

Several review papers have summarized the evolution of 3D point cloud semantic segmentation and proposed taxonomies that help contextualize our contributions. Xie et al. [24] categorize existing approaches into point-based, voxel-based, and multi-view projection families, emphasizing the trade-off between geometric fidelity and computational efficiency. More recent surveys, including Yang et al. [25] and Betsas et al. [26], extend this taxonomy by discussing sparse convolutional frameworks, hybrid fusion models, transformer-based architectures, and lightweight, attention-driven designs targeting real-time applications. According to these taxonomies, LVCA-Net falls into the voxel-based sparse convolution category while incorporating coordinate-aware attention to promote feature consistency. Unlike computationally intensive transformer-based models, our method emphasizes lightweight design and efficient spatial reasoning, aligning with the lightweight segmentation trends reported in recent surveys.

### 2.1. Point Cloud Segmentation Landscape and Voxel Discretization

Voxelization regularizes large outdoor point clouds and enables sparse convolution that is amenable to efficient hardware execution [27]. MinkUNet learns scalable 3D features through generalized sparse convolutions [16], while SPVCNN couples sparse 3D encoding with efficient decoding to control activation density [18]. Cylinder3D replaces cubic partitioning with cylindrical voxels to balance range-dependent point density, and employs asymmetric residual blocks with context separation to capture long-range dependencies [17].

Other design routes further illustrate the core trade-offs between accuracy and throughput. Point-based networks such as PointNet, PointNet++, KPConv, and RandLA-Net retain geometric fidelity but face scalability limits on large-scale urban scans [13,14,15,28]. Hybrid polar or sector mappings, such as PolarNet, aim to balance per-point precision with structured processing [29]. Range-image projection methods, including RangeNet53++ and SalsaNext, deliver high throughput but may suffer from geometric distortion and depth ambiguity [30,31]. Image-domain backbones provide strong 2D priors that continue to influence 3D perception [32,33,34].

Beyond LiDAR-only modeling, multimodal feature integration has also been explored to leverage complementary sensing modalities. For example, BEVFusion [35] unifies LiDAR and camera features in a shared bird’s-eye-view representation, which can improve robustness under occlusion and illumination changes. Although such multimodal fusion frameworks demonstrate strong performance, their objectives differ from the single-sensor, LiDAR-only semantic segmentation setting addressed in this paper.

Our method retains the voxel-based pipeline for efficiency while addressing typical discretization failures, such as boundary leakage and mixed occupancy, under limited resolution. We do so with three components: anisotropic depthwise residual units that sharpen directional geometry at low computational cost; a stride-based LiteDown–LiteUp path that preserves structural continuity through skip alignment; and coordinate-aware attention that reweights features along the radial, azimuthal, and vertical axes to restore inter-voxel consistency.

### 2.2. Lightweight 3D Network Design

Compact operators and principled model scaling are essential for real-time LiDAR segmentation. Depthwise separable convolutions and pointwise bottlenecks reduce parameters by decoupling spatial aggregation from channel projection [36,37]. In sparse 3D settings, architectures such as SPVCNN emphasize compute reuse and low activation density [18], while compound width–depth scaling strategies from 2D vision, such as EfficientNet, guide balanced scaling under tight memory budgets [34].

Transformer-based designs have also been explored to improve representational capacity under constrained computational budgets. HM-ViT [38] introduces hetero-modal Transformers for efficient feature interaction across agents and sensing perspectives, suggesting that structured attention mechanisms can enhance representation quality. Although HM-ViT focuses on cooperative perception rather than standalone LiDAR semantic segmentation, its architectural insights motivate efficient feature-mixing strategies that are relevant to lightweight 3D design.

Our LADR extends separability to 3D anisotropy by using oriented depthwise branches along the height, width, and depth axes, followed by channel projection. The LiteDown–LiteUp blocks act as controlled low-pass operators that compress and recover multi-scale context without resorting to dense tensor conversions.

### 2.3. Attention Mechanisms

Channel and spatial attention can improve feature selection; however, standard 2D formulations are insensitive to 3D anisotropy and range-driven sparsity [39,40]. Axial attention factorizes global interactions along image axes to reduce complexity [41], and range-view transformers demonstrate context modeling under structured projections [19]. Outdoor LiDAR point clouds, when organized into cylindrical voxels, exhibit directionally skewed dependencies induced by sensor geometry. We therefore introduce a Coordinate-Guided Sparse Semantic Module that pools along the radial, azimuthal, and vertical directions and applies grouped reweighting, thereby aligning attention with sensor-aware anisotropy while maintaining computational sparsity.

Recent work on feature alignment provides additional insight into structured attention. AgentAlign [42] proposes a guided correspondence mechanism to achieve consistent representation alignment across heterogeneous intelligent agents. While AgentAlign addresses a different task—cross-agent feature alignment—it motivates the broader goal of interpretable, geometry-aware attention mechanisms, which we realize through coordinate-guided reweighting in CoSSM.

Similarly, recent studies in intelligent driving suggest that attention mechanisms can enhance the utilization of key discriminative features [43]. Moreover, global–local spatio-temporal attention fusion has been shown to strengthen cross-scale contextual modeling and feature refinement in driver-state understanding tasks [44], supporting the need for more structured and geometry-aware attention in 3D perception.

Complementary to the above single-agent LiDAR segmentation approaches, recent work has explored heterogeneous collaborative perception frameworks that enable information sharing among multiple agents in autonomous systems. Representative examples include the extensible open heterogeneous perception framework proposed in [45]. These methods, however, focus on cross-agent communication and collaborative feature aggregation, which differ from the single-agent LiDAR segmentation setting addressed in this paper.

## 3. Methodology

### 3.1. LVCA-Net

**Figure 2:**
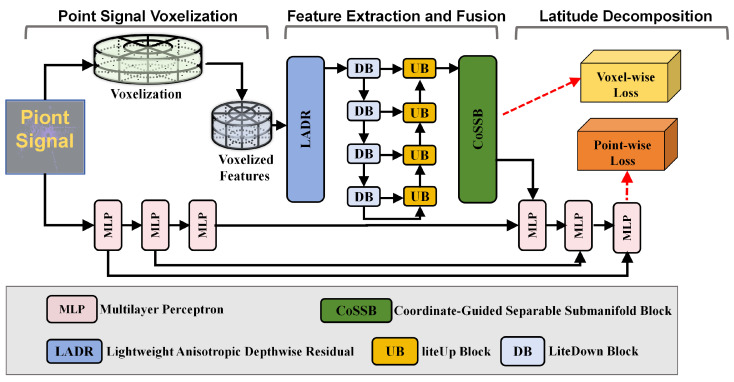
Overall framework of LVCA-Net.

To achieve high-fidelity yet efficient LiDAR segmentation in large-scale urban scenes, we propose LVCA-Net, a voxel-based coordinate-attention framework (Figure 2). Raw point clouds are first transformed from Cartesian to cylindrical coordinates and discretized into a sparse voxel grid, where the cylindrical representation helps normalize point density across near and far regions. The radial bin size increases with range to mitigate long-range sparsity, yielding a more uniform occupancy distribution.The red dashed arrows in Figure 2 indicate the training-time supervision paths, corresponding to voxel-wise and point-wise losses, which provide optimization signals during backpropagation but do not participate in forward feature propagation.

The encoder–decoder backbone is built entirely on sparse 3D convolutions. Each encoder stage stacks one or two LADR modules for feature extraction, followed by a LiteDown block for resolution reduction. Strides are set to (2,2,1), (2,2,1), and (2,2,1) to preserve vertical detail while enlarging the horizontal receptive field. A lightweight CoSSM (Coordinate-Guided Sparse Semantic Module) unit is placed at low-resolution stages to inject global anisotropic dependencies with minimal overhead. The decoder mirrors the encoder using sparse inverse convolutions and skip connections to recover fine-grained geometry.

**Figure 3:**
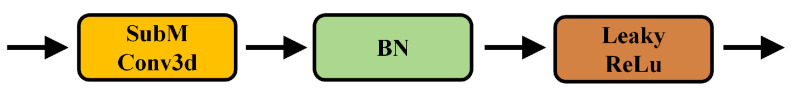
Separable submanifold convolution module (SepSubM).

A lightweight Separable Submanifold Convolution Module (SepSubM) serves as the neck, aggregating decoder features through 1×1×1 alignment and attention-guided fusion to produce a high-resolution semantic volume for the final 1×1×1 segmentation head (Figure 3). The entire pipeline remains fully sparse, avoiding dense conversions, and employs asymmetric separable kernels to improve efficiency. The framework supports both single-frame and multi-frame inputs; when multiple scans are used, late fusion is applied at the neck.

### 3.2. Lightweight Anisotropic Depthwise Residual

**Figure 4:**
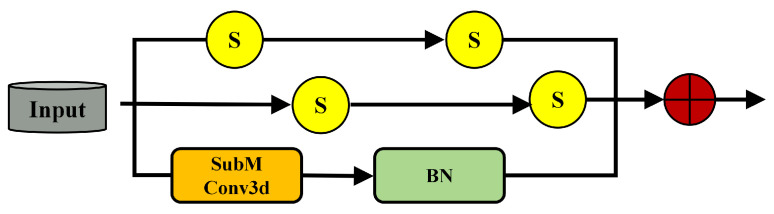
LADR architecture with anisotropic depthwise convolutions.

The Lightweight Anisotropic Depthwise Residual (LADR) serves as the primary residual unit in LVCA-Net (Figure 4). It improves the trade-off between feature representation capacity and computational efficiency. Unlike conventional 3D residual blocks, the LADR incorporates a separable submanifold bottleneck in its residual path to enable efficient feature propagation under sparsity. The same bottleneck is reused in the coordinate-guided module (CoSSM) to keep the overall architecture lightweight and coherent.

Given an input voxel tensor $X\in R^{B\times C\times D\times H\times W}$, the separable submanifold bottleneck computes:

(1) $$X^{\prime }=\sigma \;\left(\mathrm{BN}({\mathrm{Conv}}_{1\times 1\times 1}\left(X\right))\right),$$

(2) $$X^{\prime \prime }=\sigma \;\left(\mathrm{BN}({\mathrm{DWConv}}_{k\times k\times k}\left(X^{\prime }\right))\right),$$

(3) $$F=\sigma \;\left(\mathrm{BN}({\mathrm{Conv}}_{1\times 1\times 1}\left(X^{\prime \prime }\right))\right).$$

The bottleneck processes only occupied voxels, thereby reducing computation for large-scale LiDAR data.

LADR further enhances feature discrimination through three anisotropic depthwise convolutions applied along complementary spatial axes:

(4) $$F_{x}={\mathrm{DWConv}}_{1\times 3\times 3}\left(F\right),\;F_{y}={\mathrm{DWConv}}_{3\times 1\times 3}\left(F\right),\;F_{z}={\mathrm{DWConv}}_{3\times 3\times 1}\left(F\right).$$

Each branch focuses on one spatial direction: depth *D*, height *H*, or width *W*. The branch outputs are fused and projected via channel mixing:

(5) $$F_{\mathrm{agg}}=F_{x}+F_{y}+F_{z},$$

(6) $$Y=\sigma \;\left(\mathrm{BN}({\mathrm{Conv}}_{1\times 1\times 1}\left(F_{\mathrm{agg}}\right))\right),$$

A residual skip connection stabilizes optimization:

(7) Z=X+Y.

The separable bottleneck is also used in the coordinate-guided module (CoSSM), where it is combined with coordinate attention to refine multi-scale feature fusion along the radial, vertical, and azimuthal directions. This unified design promotes inter-voxel consistency and improves boundary precision in sparse LiDAR point clouds. The module provides an effective trade-off between accuracy and computational cost, supporting real-time inference in outdoor environments.

### 3.3. LiteDown Block and LiteUp Block

**Figure 5:**
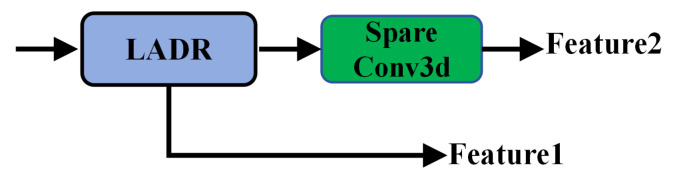
LiteDown block.

**Figure 6:**
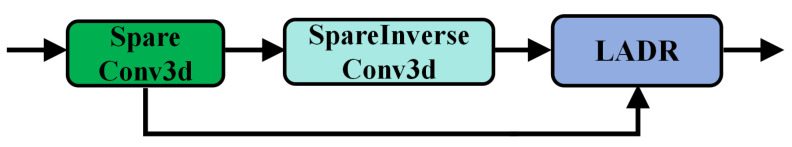
LiteUp block.

The LiteDown (Figure 5) and LiteUp (Figure 6) blocks form the main encoder–decoder transition modules of LVCA-Net. They compress and reconstruct voxel features while maintaining semantic consistency across scales. Both modules use depthwise separable sparse convolutions to reduce computational cost while preserving geometric structure.

The LiteDown block performs spatial downsampling via stride-based depthwise convolution, reducing voxel resolution while preserving essential feature responses. Given an input tensor $X\in R^{B\times C\times D\times H\times W}$, the operation is defined as:

(8) Y=σ(BN(DWConv(X;s))).

Here, *s* denotes the stride that controls voxel resolution reduction. The depthwise operator applies independent three-dimensional filters to each channel.

(9) $$\mathrm{DWConv}\left(X;s\right)=X*K_{d},\;K_{d}\in R^{C\times 1\times k\times k\times k}.$$

This factorization decouples spatial aggregation from channel mixing. This operation can be interpreted as a low-pass filter, attenuating high-frequency noise and outliers while preserving the dominant geometric structure of LiDAR data.

A pointwise convolution is then applied to restore channel expressivity:

(10) Z=PWConv(Y),

where $\mathrm{PWConv}={\mathrm{Conv}}_{1\times 1\times 1}$. The complete LiteDown process can be expressed as:

(11) LiteDown(X)=PWConv(σ(BN(DWConv(X;s)))).

This structure performs compact spatial compression and can be viewed as a factorized mapping of spatial and channel transformations. It assumes that voxel features lie on a low-dimensional manifold and thus encourages low reconstruction error:

(12) $$\min_{\hat{X}}{∥X-U\left(D\left(X\right)\right)∥}_{2}^{2},$$

where D and U represent the LiteDown and LiteUp mappings. The downsampling serves as an information bottleneck, enhancing compactness and robustness.

The LiteUp block reverses this process via sparse transposed convolution and feature refinement. It reconstructs spatial details by upsampling voxel features from coarser scales and merging them with encoder skip connections:

(13) $$\hat{X}=\mathrm{BN}\left(\mathrm{PWConv}\left(\mathrm{DWDeconv}\left(X\right)\right)\right).$$

Here, DWDeconv denotes depthwise transposed convolution, which restores voxel resolution while maintaining computational sparsity.

Through the symmetric design of LiteDown and LiteUp, LVCA-Net performs efficient feature compression and reconstruction, helping maintain geometric continuity and accurate semantic recovery across multiple scales in large outdoor scenes.

### 3.4. Coordinate-Guided Sparse Semantic Module

**Figure 7:**
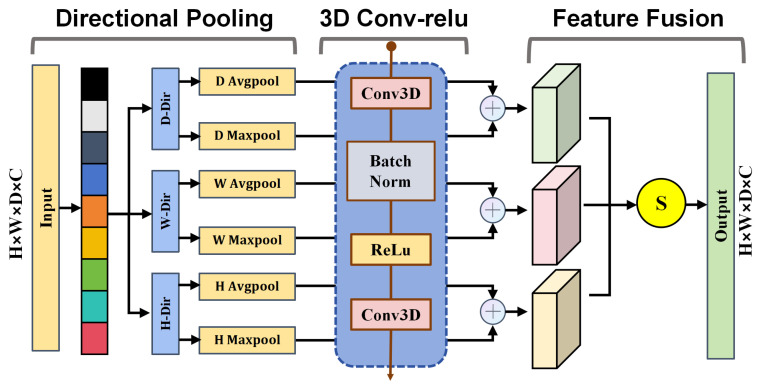
Coordinate-Guided Sparse Semantic Module.

To complement local feature extraction in the LADR and LiteDown modules, we propose the Coordinate-Guided Sparse Semantic Module (CoSSM). This module enhances the decoder’s feature representation capability by embedding coordinate-guided attention within a sparse separable bottleneck. Unlike conventional convolutional fusion layers that treat all spatial regions uniformly, CoSSM adaptively adjusts feature responses along the radial, azimuthal, and vertical dimensions in cylindrical voxel space, so that geometrically distinct regions receive appropriate attention weights. The overall structure is shown in Figure 7.

Given an input voxel tensor $X\in R^{B\times C\times D\times H\times W}$, CoSSM divides the feature channels into *G* groups:

(14) $$X=\left[X_{1},X_{2},\ldots ,X_{G}\right],\;X_{g}\in R^{B\times (C/G)\times D\times H\times W}.$$

For each group $X_{g}$, spatial pooling is performed along three coordinate dimensions to obtain directional context descriptors:

(15) $$A_{h}={\mathrm{AvgPool}}_{h}\left(X_{g}\right)+{\mathrm{MaxPool}}_{h}\left(X_{g}\right),$$

(16) $$A_{w}={\mathrm{AvgPool}}_{w}\left(X_{g}\right)+{\mathrm{MaxPool}}_{w}\left(X_{g}\right),$$

(17) $$A_{d}={\mathrm{AvgPool}}_{d}\left(X_{g}\right)+{\mathrm{MaxPool}}_{d}\left(X_{g}\right).$$

Each descriptor is processed through a shared 1×1×1 convolution to generate directional attention weights:

(18) $$\alpha_{h}=\sigma \left(\mathrm{Conv}\left(A_{h}\right)\right),\;\alpha_{w}=\sigma \left(\mathrm{Conv}\left(A_{w}\right)\right),\;\alpha_{d}=\sigma \left(\mathrm{Conv}\left(A_{d}\right)\right),$$

Here, σ denotes the activation function. The reweighted feature for each group is computed as:

(19) $$Y_{g}=X_{g}⊗\left(\alpha_{h}\cdot \alpha_{w}\cdot \alpha_{d}\right),$$

The aggregated feature is obtained as:

(20) $$Y=\mathrm{Concat}(Y_{1},Y_{2},\ldots ,Y_{G}).$$

The enhanced feature *Y* is then passed through the separable submanifold bottleneck to refine inter-channel relationships while maintaining computational sparsity:

(21) Z=PWConv(BN(DWConv(Y))).

This sequential integration of CoSSM and SepSubM enables CoSSM to balance coordinate-guided attention and efficient sparse convolution, promoting fine-grained feature aggregation and boundary precision. By coupling spatial attention with lightweight convolution, CoSSM enhances multi-scale semantic fusion in LVCA-Net while maintaining low latency and parameter efficiency for large-scale LiDAR perception.

## 4. Experiment and Analysis

In this section, we benchmark the proposed model across multiple evaluation settings. For semantic segmentation, we evaluate the proposed method on two widely used large-scale benchmarks, namely SemanticKITTI and nuScenes. In addition, we conduct extensive ablation studies to quantify the contribution of each component, and perform robustness tests, transferability experiments, resolution analyses, and loss-function evaluations to provide a comprehensive assessment of the proposed framework.

### 4.1. Experimental Setup

All experiments were conducted on a single computing platform to ensure controlled and reproducible results. The software environment consisted of Ubuntu 20.04 LTS, PyTorch 1.10.1, and Python 3.8.19. The hardware configuration included an Intel Core i7-14700KF CPU and an NVIDIA GeForce RTX 3090 GPU, providing sufficient computational resources for training and inference. This setup provides stable runtime performance and reliable benchmarking across different datasets and settings. The final hyperparameter configurations used in all experiments are summarized in Table 1. Voxel-based methods such as Cylinder3D, SPVCNN, and MinkUNet were retrained on our platform using the same training protocol as LVCA-Net. In contrast, large-capacity transformer-based models (PTv2, PTv3, UniSeg) require training resources that exceed our available hardware, and therefore their results are reported directly from the official numbers published in their respective papers. This distinction is explicitly noted to ensure a transparent and consistent comparison across all methods.

In addition to the software and hardware environment, we report the computational complexity and runtime characteristics of LVCA-Net to clarify its efficiency profile. The complete model contains 4.6 M parameters and requires approximately 15.4 GFLOPs per frame under the default voxel resolution (480 × 360 × 32). When deployed on an NVIDIA RTX 3090 GPU, LVCA-Net achieves a real-time inference throughput of 12.5–18 fps, depending on the dataset and input configuration.

### 4.2. Evaluation Metrics and Datasets

#### 4.2.1. Evaluation Metrics

To comprehensively evaluate LVCA-Net, we adopted three widely used metrics in LiDAR semantic segmentation:

- mIoU (Mean Intersection over Union): The average IoU across all semantic classes, reflecting overall segmentation quality.
- Acc (Overall Accuracy): The proportion of correctly classified points among all points.
- Acc_cls (Class-wise Accuracy): The mean accuracy across all categories, providing a balanced evaluation of per-class performance.

#### 4.2.2. Datasets

We evaluated our method on two authoritative large-scale benchmarks:

- SemanticKITTI: A LiDAR dataset derived from KITTI odometry sequences, providing dense, point-wise labels for 22 sequences (19 classes used for training/testing). We followed the official train/validation/test splits and reported both single-frame and multi-frame results.
- nuScenes: A large-scale autonomous driving dataset containing 1000 driving scenes with 16 semantic classes. Following the nuScenes lidarseg benchmark, we report results on 16 semantic classes.

These two datasets complement each other: SemanticKITTI emphasizes sequential LiDAR scans in suburban/urban roads, while nuScenes highlights diverse traffic conditions in dense urban centers.

### 4.3. Loss Function

Following common practice, the final loss function consists of a voxel-wise segmentation loss and a point-wise refinement term. Specifically, voxel predictions are supervised using a weighted cross-entropy loss:

(22) $$L_{\mathrm{voxel}}=-\sum_{i=1}^{N}w_{y_{i}}\log p\left(y_{i}|x_{i}\right),$$

where $w_{y_{i}}$ is the class weight used to address class imbalance. To mitigate voxel quantization errors, we refine point-level predictions by projecting voxel features back to the original points and optimizing them with a standard cross-entropy loss:

(23) $$L_{\mathrm{point}}=-\sum_{j=1}^{M}\log p\left(y_{j}|p_{j}\right).$$

The final objective is defined as a weighted sum:

(24) $$L=L_{\mathrm{voxel}}+\lambda L_{\mathrm{point}},$$

where λ controls the relative contribution of voxel-level and point-level supervision. This hybrid design helps alleviate semantic ambiguity introduced by voxelization and improves fine-grained segmentation accuracy.

### 4.4. Comparative Experiment

#### 4.4.1. Results on SemanticKITTI (Single-Frame)

We first evaluate LVCA-Net on the SemanticKITTI dataset under single-frame settings. As summarized in Table 2, our model achieves an mIoU of 67.17%, Acc of 91.79%, and Acc_cls of 74.19%, outperforming all voxel-based and hybrid baselines while maintaining a lower parameter count. These results demonstrate that LVCA-Net achieves a superior trade-off between segmentation accuracy and computational efficiency.

Compared with voxel-based baselines such as Cylinder3D (62.14% mIoU) and MinkUNet (62.31% mIoU), LVCA-Net delivers an improvement of approximately 5 percentage points in mIoU. It also surpasses hybrid frameworks such as SPVCNN (62.33% mIoU) and RPVNet (63.8% mIoU), suggesting that the introduction of coordinate-aware attention (CoSSM) and lightweight asymmetric convolution (LADR) can enhance feature expressiveness under sparse LiDAR conditions. Importantly, LVCA-Net achieves these improvements with limited computational overhead, making it well suited for real-time inference in autonomous driving applications.

In terms of class-wise IoUs, LVCA-Net shows advantages in categories characterized by long-range sparsity and thin geometric structures, such as Truck (87.86%), Person (81.27%), and Bicyclist (87.12%), where voxel quantization can degrade feature quality. For dense and static categories including Building, Road, and Vegetation, the network maintains high accuracy (e.g., 94.74% IoU on Road), indicating a balanced ability to capture both local geometric details and global contextual semantics.

**Figure 8:**
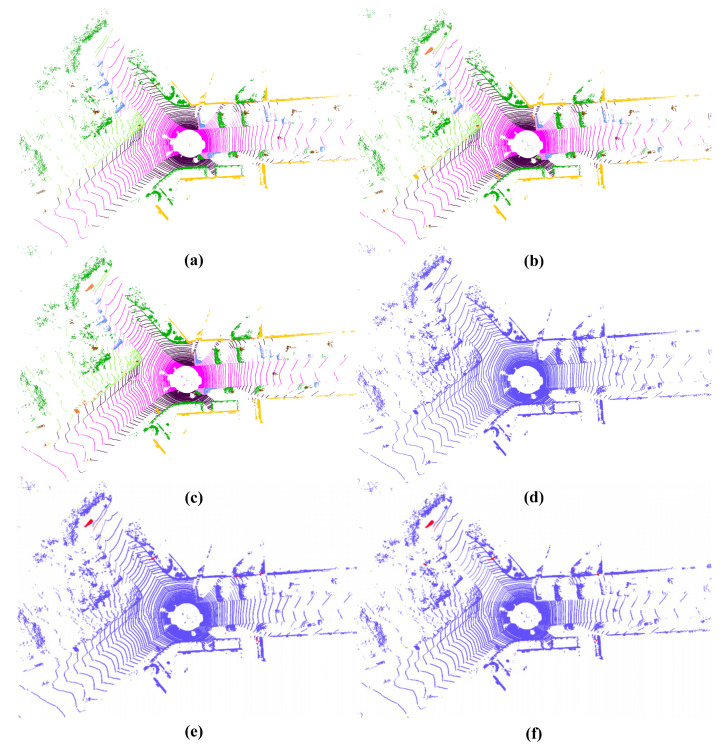
Visualization results on SemanticKITTI. (**a**) Ground truth. (**b**) Cylinder3D. (**c**) LVCA-Net. (**d**) Raw point cloud. (**e**) Difference (Cylinder3D vs. ground truth). (**f**) Difference (LVCA-Net: vs. ground truth).

Overall, these quantitative results (see Table 2 and Table 3) indicate that LVCA-Net improves segmentation precision while maintaining strong generalization ability. The qualitative visualizations in Figure 8 further support this observation: compared with Cylinder3D, LVCA-Net produces more complete object boundaries, fewer false positives (shown in red), and higher semantic consistency with the ground truth, especially in regions with sparse or occluded points.

To further benchmark the lightweight characteristics of LVCA-Net, we compare it with several recent transformer-based state-of-the-art LiDAR segmentation frameworks, including PTv2, PTv3, and UniSeg. Early transformer-based designs such as PTv1 [51] suggest the benefit of global receptive fields in LiDAR segmentation, while later variants (PTv2/PTv3) substantially scale up model depth and width to maximize accuracy. These models, however, fall into the category of large-capacity architectures, achieving strong segmentation performance at the cost of significantly increased computational and memory requirements.

When the input point clouds are spatially cropped or sparsified, large-capacity models such as PTv1 and PTv2 can be executed on lower-power hardware platforms. However, fully exploiting their representational capacity and reported peak performance typically requires high-end computational resources. Given that such conditions cannot be fully satisfied on our experimental platform, we therefore report the officially published results from the original papers for these large-scale models. As shown in Table 4, LVCA-Net achieves competitive segmentation accuracy with only 4.6 M parameters, which is substantially smaller than PTv2 (12.8 M), PTv3 (46.2 M), and UniSeg (147.6 M). Although its mIoU is slightly lower than these high-capacity models, the significant reduction in model size reflects a more favorable accuracy–efficiency trade-off, making LVCA-Net particularly suitable for real-time perception and deployment on resource-constrained intelligent machines.

#### 4.4.2. Results on SemanticKITTI (Multi-Frame)

In accordance with the SemanticKITTI multi-scan protocol, we adopt a three-frame fusion strategy in our multi-frame experiments. For each target frame at time *t*, two preceding LiDAR scans at times t−1 and t−2 are loaded (multiscan = 2), rigidly transformed into the target coordinate system using ground-truth poses, and concatenated at the point level. This yields a fused three-frame input, which is subsequently voxelized using the same cylindrical grid configuration.

As presented in Table 5, LVCA-Net achieves the best overall performance on the SemanticKITTI multi-frame benchmark, reaching 51.44% mIoU and outperforming the compared voxel- and point-based baselines. Compared with KPConv, which attains 51.2% mIoU but relies on dense point convolutions with substantial computational costs, LVCA-Net achieves comparable (or higher) accuracy while maintaining a lightweight voxelized structure.

LVCA-Net shows advantages on categories characterized by sparsity or high motion variance, including Bicycle (57.0%), Motorcycle (77.0%), and Truck (44.0%), with notable gains over KPConv. These results suggest that coordinate-aware attention (CoSSM) can strengthen geometric continuity and capture anisotropic dependencies across temporal frames.

In static object classes such as Building, Vegetation, and Terrain, LVCA-Net maintains competitive or superior accuracy (91.0%, 85.0%, and 69.0%, respectively), suggesting that it preserves fine structural features while remaining efficient. Furthermore, the model improves on moving-object categories such as Mov Person (64.0%) and Mov Bicyclist (62.0%), supporting the benefit of temporal voxel aggregation for dynamic perception.

Overall, these results indicate that LVCA-Net achieves a strong balance between precision, efficiency, and robustness in multi-frame 3D LiDAR segmentation, with clear advantages in both sparse and dynamic environments.

#### 4.4.3. Results on nuScenes

As presented in Table 6, on the nuScenes dataset, LVCA-Net achieves an mIoU of 77.1%, outperforming voxel-based baselines such as Cylinder3D (73.3%) and MSF-CSCNet (75.2%). Despite the sparse point distribution and uneven class distribution in nuScenes, LVCA-Net maintains consistent segmentation performance on distant and thin-structured objects such as bicycle, traffic cone, and vegetation.

The performance gains of LVCA-Net can be attributed to its capacity for joint spatial–geometric modeling and semantic coherence. Anisotropic depthwise operations enhance geometric sensitivity along principal spatial directions, facilitating stable pattern extraction under varying scales and point densities. An adaptive hierarchical encoder–decoder structure manages voxel sparsity while maintaining cross-scale contextual integrity. Additionally, a coordinate-group attention mechanism reinforces directional dependencies during feature interaction in 3D space, improving discrimination in regions with uneven density and enhancing boundary precision in complex urban scenes. structures such as intersections and facades.

### 4.5. Noise Robustness Experiments

To examine the resilience of LVCA-Net under noisy and incomplete LiDAR inputs, we conduct controlled experiments on the SemanticKITTI dataset by injecting three types of perturbations: random point dropout, uniform noise, and Gaussian noise. These settings emulate common real-world degradations arising from reflective interference, sensor drift, and environmental disturbances.

As summarized in Table 7, LVCA-Net achieves the best performance across all evaluated noise conditions, while maintaining stable segmentation accuracy and class-wise balance. Under random dropout, its mIoU remains at 23.40%, substantially higher than Cylinder3D (14.48%) and SPVCNN (11.65%), indicating stronger robustness to missing-point artifacts. For additive noise scenarios, LVCA-Net attains 16.10% mIoU under uniform noise and 17.05% under Gaussian noise, outperforming the compared voxel-based baselines. These quantitative results suggest that LVCA-Net can retain semantic integrity even when point distributions become sparse or corrupted.

**Figure 9:**
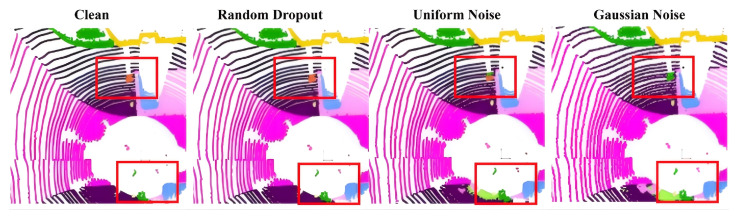
Robustness evaluation of LVCA-Net under three noise conditions (random point dropout, uniform noise, and Gaussian noise) on the SemanticKITTI dataset. (Red boxes indicate regions where segmentation differences are most evident under different noise perturbations).

The qualitative comparison in Figure 9 further supports these findings. LVCA-Net exhibits clearer object boundaries and fewer false positives compared with the baseline models, especially in occluded or low-density regions. This robustness can be attributed to the coordinated effects of its submodules: the anisotropic convolutions in LADR enhance geometric stability; the hierarchical LiteDown/LiteUp blocks suppress high-frequency perturbations while maintaining contextual coherence; and CoSSM adaptively reweights spatial dependencies along cylindrical dimensions, reducing the risk of feature collapse.

In summary, LVCA-Net demonstrates strong robustness to diverse noise perturbations by preserving both geometric topology and semantic continuity, as supported quantitatively in Table 7 and qualitatively in Figure 9.

### 4.6. Transferability Experiments

To assess the cross-dataset generalization capability of LVCA-Net, transfer experiments are conducted between the SemanticKITTI and nuScenes datasets, which differ substantially in LiDAR beam density, sensor configuration, and environmental distribution. In this evaluation, the model is trained on SemanticKITTI and directly tested on nuScenes without any fine-tuning.

As presented in Table 8, LVCA-Net achieves the highest transfer performance, with an mIoU of 77.1% from scratch and 77.4% after pre-training. These results indicate that LVCA-Net effectively adapts to the domain shift between 64-beam and 32-beam LiDAR sensors, maintaining stable segmentation quality under different sensing configurations. Compared with Cylinder3D and SPVCNN, LVCA-Net improves mIoU by more than 3.5% while using significantly fewer parameters, achieving 4.6 MB compared with 56.8 MB and 14.7 MB. This demonstrates superior parameter efficiency and strong domain robustness.

The improvement arises from the network’s design, which combines anisotropic convolutional encoding with coordinate-aware attention to enhance feature transferability across spatial distributions. By jointly modeling voxel geometry and directional dependencies, LVCA-Net mitigates degradation caused by changes in sampling density and viewpoint distribution, enabling consistent recognition of both static structures and dynamic agents across datasets.

Overall, the results confirm that LVCA-Net maintains strong adaptability under cross-dataset evaluation and provides reliable generalization for large-scale autonomous driving perception without the need for dataset-specific retraining.

### 4.7. Loss Coefficient Experiments

To analyze the effect of balancing voxel-level and point-level supervision on segmentation performance, experiments were conducted by varying the loss coefficient λ in the overall objective function. The voxel-level loss promotes global semantic consistency through volumetric supervision, whereas the point-level loss refines local geometry by projecting voxel features back to individual points. The combined objective allows the model to jointly optimize structural awareness and boundary precision.

As shown in Table 9, the segmentation performance exhibits a clear dependency on λ. When λ is too small, the model overemphasizes voxel-level information, leading to over-smoothed predictions and reduced boundary accuracy. Conversely, excessively large values cause overfitting to local features and unstable convergence in sparse regions. The optimal balance is achieved when λ=0.9, yielding the highest mIoU of 67.17%. This configuration ensures that voxel-level supervision contributes to stable contextual modeling, while point-level guidance effectively enhances fine structural delineation.

These observations confirm that appropriate weighting between voxel- and point-level losses is crucial for achieving high segmentation accuracy and convergence stability in LVCA-Net.

### 4.8. Ablation Experiments

Table 10 presents the analysis of each component’s contribution to the performance of LVCA-Net. Starting from the voxel-based baseline with 62.14% mIoU, the addition of CoSSM increases segmentation accuracy by 2.75%, demonstrating the effectiveness of coordinate-group attention in stabilizing directional feature responses. LADR enhances performance by 3.31% through anisotropic depthwise–pointwise convolution, which captures elongated geometric cues and detailed local structures with reduced computational cost. The LiteDown and LiteUp modules improve mIoU by 2.12%, enabling efficient multiscale encoding and decoding that preserve structural continuity and boundary precision.

The integration of multiple modules produces a more stable and consistent improvement. Combining CoSSM and LADR yields 66.14% mIoU, demonstrating that attention-guided normalization and anisotropic filtering correct complementary types of voxel feature ambiguity. Incorporating LADR with LiteDown–LiteUp achieves 66.51%, indicating that directional feature extraction benefits from hierarchical aggregation and enhanced multiscale representation. The configuration combining CoSSM with LiteDown–LiteUp attains the highest overall accuracy and class-wise consistency, reflecting balanced feature calibration through adaptive attention and multiscale fusion.

With all three components activated, the full model reaches 67.17% mIoU, 91.79% overall accuracy, and 74.19% class-wise accuracy. These findings verify that CoSSM, LADR, and the lightweight encoder–decoder architecture contribute complementary effects: CoSSM enhances global spatial awareness, LADR strengthens directional discrimination, and the encoder–decoder pathway maintains scale robustness while suppressing over-smoothing in sparse or noisy regions.

### 4.9. Statistical Significance Analysis

To ensure the reliability and reproducibility of the reported performance, we conduct a statistical variation study following a commonly used evaluation protocol in 3D semantic segmentation. All models, including LVCA-Net and representative baselines (Cylinder3D and SPVCNN), are trained and evaluated five times using different random seeds. We report the mean and standard deviation (mean ± std) of mIoU and overall accuracy, as well as the corresponding 95% confidence intervals (CI).

Table 11 summarizes the statistical results on the SemanticKITTI single-frame benchmark. LVCA-Net achieves consistently higher performance across all runs with low variance, indicating stable convergence behavior. In contrast, the baseline models show larger fluctuations, suggesting higher sensitivity to initialization or sampling randomness.

To further assess the statistical reliability of the observed improvements, we perform paired *t*-tests between LVCA-Net and the two baselines. The resulting *p*-values are below 0.01 for both comparisons, indicating that the performance gains of LVCA-Net are statistically significant and unlikely to arise from random variation. This result supports the conclusion that the proposed architectural enhancements—including anisotropic depthwise residual modules, lightweight encoder–decoder pathways, and coordinate-guided semantic attention—provide robust performance benefits beyond stochastic training effects.

Overall, the statistical analysis indicates that LVCA-Net consistently outperforms existing voxel-based architectures with statistically significant gains, reinforcing the robustness and reliability of the improvements reported in this study.

## 5. Discussion

LVCA-Net improves LiDAR semantic segmentation by enhancing accuracy, robustness, and computational efficiency on large-scale benchmarks. On the SemanticKITTI dataset, it achieves 67.17% mIoU with balanced class-wise performance, remains stable under multiple noise perturbations, and exhibits strong transferability across sensors with different resolutions while using substantially fewer parameters than other voxel-based baselines. These results suggest that the proposed design alleviates the long-standing trade-off between geometric precision and efficiency observed in point-based, voxel-based, and hybrid methods.

Ablation analysis highlights the complementary effects of the main components. The CoSSM module stabilizes directional dependencies in the cylindrical space, mitigating density imbalance and improving recognition of elongated or sparsely sampled structures. The LADR module refines geometric features through depthwise–pointwise convolutions, improving local details while maintaining low computational costs. The LiteDown–LiteUp pathway supports multiscale feature fusion without heavy decoders, helping preserve boundaries and suppress noise. Together, these modules help reduce voxel-quantization blur while maintaining global contextual integrity.

LVCA-Net also demonstrates strong resilience and generalization. Under random dropout, uniform noise, and Gaussian noise, its performance degradation is smaller than that of competing baselines, suggesting that coordinate-aware attention and anisotropic convolution contribute to structural stability. In cross-dataset transfer, the network maintains high accuracy without retraining, supporting its adaptability to different sensor configurations and scene distributions.

The model achieves a favorable balance between accuracy and latency while maintaining interpretability and structural simplicity. Future work may focus on explicit domain adaptation and refined temporal modeling for multi-frame data. Performance under extremely sparse inputs or adverse weather conditions remains a potential limitation that warrants further exploration.

In summary, LVCA-Net integrates coordinate-aware attention, anisotropic residual structures, and lightweight multiscale aggregation into a unified, efficient framework for 3D LiDAR perception. Its design supports stable performance across varying density, noise, and sensor conditions, making it well suited for deployment in real-world autonomous driving systems.

## 6. Conclusions

This study presents LVCA-Net, a lightweight voxel–coordinate attention framework for accurate and efficient LiDAR semantic segmentation in complex urban environments. The proposed architecture integrates four complementary components: an anisotropic depthwise residual module (LADR) for direction-aware geometric feature extraction, a LiteDown–LiteUp encoder–decoder pathway for hierarchical representation learning, a Coordinate-Guided Sparse Semantic Module (CoSSM) for sensor-aware attention in the cylindrical voxel space, and a lightweight SepSubM neck for multi-scale feature aggregation. All modules are implemented using sparse operations, enabling LVCA-Net to maintain low computational costs while preserving geometric fidelity.

Extensive experimental evaluations demonstrate that LVCA-Net achieves a strong balance between accuracy, robustness, and efficiency across multiple benchmarks. On the SemanticKITTI dataset, the model attains 67.17% mIoU, 91.79% overall accuracy, and 74.19% class-wise accuracy, showing competitive performance among voxel-based LiDAR segmentation methods. In the multi-frame setting, LVCA-Net reaches 51.44% mIoU, indicating improved temporal consistency and enhanced recognition of dynamic objects. On the nuScenes benchmark, the model achieves 77.1% mIoU with a compact parameter size, highlighting its robustness to sparse and long-range point cloud distributions. Additional experiments on noise perturbation, cross-dataset transfer, and ablation analysis further confirm the stability of the proposed design and the complementary contributions of each architectural component, as well as the effectiveness of combining voxel-level and point-level supervision.

Overall, LVCA-Net provides a practical and scalable solution for LiDAR-only 3D semantic segmentation, making it particularly suited to real-time perception and deployment on resource-constrained intelligent machines. Future work will explore adaptive cylindrical voxelization strategies, lightweight temporal modeling for multi-scan perception, domain adaptation across heterogeneous datasets, and hardware-aware optimization for embedded platforms. Moreover, extending the framework to multi-modal perception, such as LiDAR–camera fusion, represents a promising direction for further improving scene understanding in autonomous driving systems.

## Figures and Tables

**Table 1:** Experimentalhyperparameter settings.

Hyperparameter	Value
Optimizer	Adam
Learning rate	0.001
Momentum	0.1
Weight decay	$1\times 10^{-4}$
Batch size	2
Training epochs	36
Voxel grid size	480×360×32

**Table 2:** Single-frame validation results on the SemanticKITTI benchmark (Part 1).

Method	mIoU	Acc	Acc_cls	Car	Bicycle	Motorcycle	Truck	Bus	Person	Bicyclist	Motorcyclist	Road
MinkUnet [16]	62.31	**91.88**	68.94	96.67	18.14	63.26	81.79	63.94	66.30	80.69	0	93.44
Cylinder3D [17]	62.14	91.08	66.98	96.20	28.80	62.80	89.00	56.50	73.9	0.858	0	93.90
SPVCNN [18]	62.33	91.84	69.50	96.60	25.68	61.33	73.29	61.55	68.33	0.829	0	93.15
PoinPainting [46]	54.50	85.10	61.40	94.70	17.70	35.00	28.80	60.81	59.40	63.60	0	**95.30**
SCFusion [47]	58.10	88.70	65.00	91.80	36.00	41.00	46.50	60.74	55.00	56.00	0	93.80
RPVNetn [48]	63.75	85.50	70.00	88.50	**62.10**	62.40	40.10	62.37	68.80	67.50	0	84.70
2DPASS [49]	64.61	91.56	72.87	96.74	56.88	67.14	65.20	62.85	80.03	87.54	3.54	94.21
SPVNAS [19]	60.90	85.70	67.20	89.30	47.20	67.20	83.50	60.59	70.40	83.20	0	86.90
MSF-CSCNet [50]	65.81	91.55	71.44	**96.80**	50.88	72.67	**90.36**	61.67	76.16	**90.20**	0	94.34
LVCA-Net	**67.17**	91.79	**74.19**	96.49	57.40	**77.58**	87.86	**65.65**	**81.27**	87.12	**11.70**	94.74

**Table 3:** Single-frame validation results on the SemanticKITTI benchmark (Part 2).

Method	mIoU	Acc	Acc_cls	Parking	Sidewalk	Other-Ground	Building	Fence	Vegetation	Trunk	Terrain	Pole	Traffic-Sign
MinkUnet [16]	62.31	**91.88**	68.94	48.18	80.84	0.69	90.54	61.15	87.95	67.61	74.08	62.87	45.64
Cylinder3D [17]	62.14	91.08	66.98	19.80	79.80	0	89.90	57.70	87.30	62.60	72.90	**66.50**	47.00
SPVCNN [18]	62.33	91.84	69.50	48.36	79.90	0.31	**90.84**	62.32	**88.14**	67.71	**74.13**	62.70	47.11
PoinPainting [46]	54.50	85.10	61.40	39.90	80.20	0.40	87.50	55.10	87.81	67.00	72.90	61.80	36.50
SCFusion [47]	58.10	88.70	65.00	39.00	80.19	0.30	89.20	51.70	87.80	62.00	73.70	57.10	32.30
RPVNetn [48]	63.75	85.50	70.00	**63.80**	80.65	0.302	84.80	**65.40**	87.67	**68.10**	65.10	58.80	**55.70**
2DPASS [49]	64.61	91.56	72.87	41.84	81.16	0.2841	90.39	60.77	87.42	66.78	71.43	64.32	50.60
SPVNAS [19]	60.90	85.70	67.20	41.60	80.61	1.00	83.40	54.80	87.37	61.50	65.90	60.50	47.40
MSF-CSCNet [50]	65.81	91.55	71.44	44.82	81.25	0.45	90.30	59.17	86.90	66.50	71.31	65.45	51.12
LVCA-Net	**67.17**	91.79	**74.19**	43.17	**82.03**	**1.23**	89.19	54.34	87.65	66.95	73.19	66.35	52.34

**Table 4:** Comparison with large-scale LiDAR segmentation models on SemanticKITTI (single-frame).

Method	mIoU (%)	Parameters (M)	Latency (ms)
PTv2 [52]	70.3	12.8	213
PTv3 [53]	**72.3**	46.2	**45**
UniSeg [54]	67.2	147.6	145
**LVCA-Net**	67.17	**4.6**	60

**Table 5:** Comparison of LVCA-Net and other state-of-the-art LiDAR segmentation methods on the SemanticKITTI multi-frame dataset.

Method	mIoU	Car	Bicycle	Motorcycle	Truck	Other-Vehicle	Person	Road	Parking	Sidewalk	Other-Ground	Building	Fence	Vegetation	Trunk	Terrain	Pole	Traffic	Mov Car	Mov Truck	Mov Other	Mov Person	Mov Bicyclist	Mov Motorcyclist
TangentConv [55]	34.1	84.9	2.0	18.2	21.1	18.5	1.6	83.9	38.3	64.0	15.3	85.8	49.1	79.5	43.2	56.7	36.4	31.2	40.3	1.1	6.4	1.9	30.1	42.2
DarkNet53 [56]	41.6	84.1	30.4	32.9	20.0	20.7	7.5	**91.6**	**64.9**	**75.3**	27.5	85.2	56.5	78.4	50.7	64.8	38.1	53.3	61.5	14.1	15.2	0.2	28.9	37.8
Spsequencenet [57]	43.1	88.5	24.0	26.2	29.2	22.7	6.3	90.1	57.6	73.9	27.1	**91.2**	**66.8**	84.0	66.0	65.7	50.8	48.7	53.2	**41.2**	**26.2**	36.2	2.3	0.1
KPConv [15]	51.2	**93.7**	44.9	47.2	42.5	38.6	**21.6**	86.5	58.4	70.5	26.7	90.8	64.5	84.6	70.3	66.0	57.0	53.9	**69.4**	0.5	0.5	**67.5**	**67.4**	**47.2**
**LVCA-Net**	**51.44**	90.0	**57.0**	**77.0**	**44.0**	**39.0**	17.0	90.0	62.0	74.0	**34.0**	91.0	64.0	**85.0**	**72.0**	**69.0**	**63.0**	**60.0**	66.0	1.0	2.0	64.0	62.0	2.0

**Table 6:** Results of our proposed LVCA-Net and other LiDAR segmentation methods on the nuScenes dataset.

Method	mIoU	Barrier	Bicycle	Bus	Car	Vehicle	Motorcycle	Pedestrian	Traffic Cone	Trailer	Truck	Surface	Flat	Sidewalk	Terrain	Manmade	Vegetation
RangeNet++ [30]	65.5	66.0	21.3	77.2	80.9	30.2	66.8	69.6	52.1	54.2	72.3	94.1	66.6	63.5	70.1	83.1	79.8
PolarNet [29]	71.0	74.7	28.2	85.3	90.9	35.1	77.5	71.3	58.8	57.4	76.1	96.5	71.1	74.7	74.0	87.3	85.7
SalsaNext [31]	72.2	74.8	34.1	85.9	88.4	42.2	72.4	72.2	63.1	61.3	76.5	96.0	70.8	71.2	71.5	86.7	84.4
Cylinder3D [17]	73.3	72.6	37.8	89.4	86.5	43.7	72.1	74.1	61.9	63.6	80.2	96.3	68.4	74.3	75.3	87.8	86.7
MSF-CSCNet [50]	75.2	75.1	40.3	91.3	88.3	45.8	78.7	78.7	64.5	64.1	80.9	96.5	73.2	75.0	75.6	88.1	87.3
**LVCA-Net**	**77.1**	**76.8**	**48.0**	**92.1**	**90.5**	**47.7**	**81.6**	**81.3**	**72.4**	**75.6**	**85.2**	**98.1**	**76.9**	**80.5**	**81.3**	**91.7**	**90.6**

**Table 7:** Quantitative robustness evaluation under different noise perturbations on the SemanticKITTI single-frame setting.

Method	Clean			Noise Random Dropout			Uniform Noise			Gaussian Noise		
	mIoU	Acc	Acc_cls	mIoU	Acc	Acc_cls	mIoU	Acc	Acc_cls	mIoU	Acc	Acc_cls
Cylinder3D [17]	62.14	90.32	66.98	14.48	51.99	22.21	12.42	48.19	21.02	13.06	54.76	20.54
SPVCNN [18]	62.33	91.84	69.50	11.65	45.33	20.69	12.17	47.86	19.23	12.45	50.49	19.57
MinkUNet [16]	62.31	91.88	68.94	11.16	47.05	18.45	10.47	43.83	19.19	11.01	46.16	19.14
**LVCA-Net**	**67.17**	**91.79**	**74.19**	**23.40**	**66.90**	**31.10**	**16.10**	**56.40**	**22.90**	**17.05**	**55.20**	**24.35**

**Table 8:** Transfer learning results on nuScenes (64-beam → 32-beam).

Method	mIoU from Scratch (%)	mIoU Pre-Train (%)	Params (MB)
Cylinder3D [17]	73.3	73.9	56.78
SPVCNN [18]	72.1	71.2	14.7
**LVCA-Net**	**77.1**	**77.4**	**4.6**

**Table 9:** Effect of loss coefficient λ on the segmentation performance of LVCA-Net.

**Loss Coefficient (λ)**	0.0	0.3	0.6	0.9	1.0	1.3
**mIoU (%)**	62.95	64.21	61.89	**67.17**	66.30	63.92

**Table 10:** Single-frame ablation results of LVCA-Net on SemanticKITTI.

CoSSM	LADR	LiteDown & LiteUp Block	mIoU (%)	Acc (%)	Acc_cls (%)
			62.14	91.08	66.98
✓			64.89	91.67	70.78
	✓		65.45	91.80	73.45
		✓	64.26	91.06	71.01
✓	✓		66.14	91.63	73.32
✓		✓	65.81	91.81	74.36
	✓	✓	66.51	91.57	73.68
✓	✓	✓	67.17	91.79	74.19

**Table 11:** Single-frame statistical variation analysis on SemanticKITTI (5 runs).

Method	mIoU (Mean ± Std)	Acc (Mean ± Std)	Acc_cls (Mean ± Std)	95% CI (mIoU)
Cylinder3D [17]	62.14 ± 0.21	90.32 ± 0.18	66.98 ± 0.25	[61.95, 62.33]
SPVCNN [18]	62.33 ± 0.27	91.84 ± 0.20	69.50 ± 0.22	[62.07, 62.59]
**LVCA-Net**	**67.17 ± 0.19**	**91.79 ± 0.16**	**74.19 ± 0.18**	**[67.00, 67.34]**

## Data Availability

All datasets used in this study are publicly available. The SemanticKITTI dataset can be accessed at https://semantic-kitti.org/(accessed on 12 March 2024), and the nuScenes-lidarseg dataset is available at https://www.nuscenes.org/lidarseg (accessed on 20 August 2024). To ensure full reproducibility, all source code, pretrained models, configuration files, and experiment scripts have been released at: https://github.com/Spiritual-Jade/LVCA-Net.git.
